# Single-cell and ensemble activity of lumbar intermediate and ventral horn interneurons in the spinal air-stepping cat

**DOI:** 10.1152/jn.00202.2021

**Published:** 2021-12-01

**Authors:** Chantal McMahon, David P. Kowalski, Alexander J. Krupka, Michel A. Lemay

**Affiliations:** ^1^School of Biomedical Engineering, Science and Health Systems, Drexel University, Philadelphia, Pennsylvania; ^2^Department of Biology, DeSales University, Center Valley, Pennsylvania; ^3^Department of Bioengineering, Temple University, Philadelphia, Pennsylvania

**Keywords:** central pattern generator, modularity, motor primitives, neural ensemble, spinal cord injury

## Abstract

We explored the relationship between population interneuronal network activation and motor output in the adult, in vivo, air-stepping, spinal cat. By simultaneously measuring the activity of large numbers of spinal interneurons, we explored ensembles of coherently firing interneurons and their relation to motor output. In addition, the networks were analyzed in relation to their spatial distribution along the lumbar enlargement for evidence of localized groups driving particular phases of the locomotor step cycle. We simultaneously recorded hindlimb EMG activity during stepping and extracellular signals from 128 channels across two polytrodes inserted within lamina V–VII of two separate lumbar segments. Results indicated that spinal interneurons participate in one of two ensembles that are highly correlated with the flexor or the extensor muscle bursts during stepping. Interestingly, less than half of the isolated single units were significantly unimodally tuned during the step cycle whereas >97% of the single units of the ensembles were significantly correlated with muscle activity. These results show the importance of population scale analysis in neural studies of behavior as there is a much greater correlation between muscle activity and ensemble firing than between muscle activity and individual neurons. Finally, we show that there is no correlation between interneurons’ rostrocaudal locations within the lumbar enlargement and their preferred phase of firing or ensemble participation. These findings indicate that spinal interneurons of lamina V–VII encoding for different phases of the locomotor cycle are spread throughout the lumbar enlargement in the adult spinal cord.

**NEW & NOTEWORTHY** We report on the ensemble organization of interneuronal activity in the spinal cord during locomotor movements and show that lumbar intermediate zone interneurons organize in two groups related to the two major phases of walking: stance and swing. Ensemble organization is also shown to better correlate with muscular output than single-cell activity, although ensemble membership does not appear to be somatotopically organized within the spinal cord.

## Graphical abstract

**Figure:**
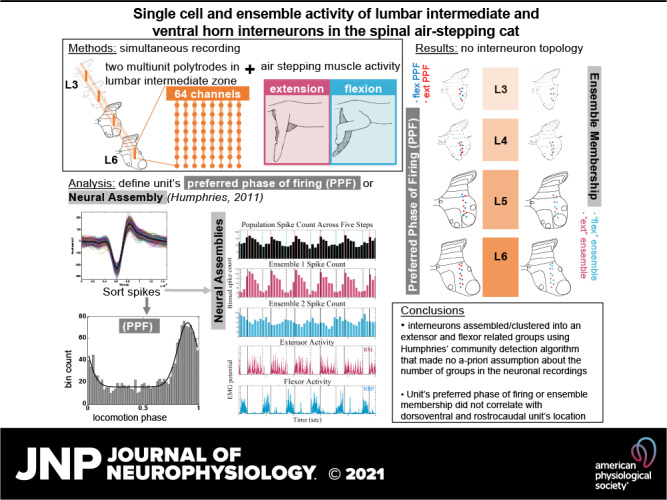

## INTRODUCTION

Spinal interneurons are constituent of the locomotor circuitry and that circuitry is intrinsically capable of maintaining and modulating rhythmic-patterned behavior. Studies in mammals show that interneurons involved in locomotor activity are for the most part located in the lumbar intermediate and ventral horns (laminae VI, VII, VIII, and X) (1–4). Centers essential to locomotion have been isolated to lumbar segments 3–4 in the cat, although caudal segments are still capable of rhythmic motor output (5, 6), studies in rodents suggest a similar distribution of rhythmogenic centers but with a rostral shift in the location of the network (7–10).

There are currently two main hypotheses on the spatial organization of the populations of lumbar spinal interneurons driving the alternating phases of stepping. One hypothesis suggests that these populations are spatially segregated within the spinal cord. Studies supporting this hypothesis demonstrate that flexor-related rhythm generation interneurons are located caudally to extensor-related interneurons (9, 11) and that subsets of V1 inhibitory interneurons settle at different locations in the cord and have different inputs (12). Further evidence of segregation comes from results in postnatal mice where premotor interneuron populations regulating antagonistic, hindlimb motor pools are spatially segregated, with flexor-related populations located ventrolaterally from extensor-related premotor populations (13). Additional findings in lamina VIII of the neonatal rat provide evidence that ipsilateral, flexor-related commissural interneurons (CINs) are located in a more ventral area than contralateral, flexor-related CINs (14).

The second hypothesis states that interneuron populations are not spatially segregated but are instead evenly distributed throughout the lumbar enlargement. Studies of embryonic and neonatal mouse spinal development indicate that networks with rhythmogenic potential are distributed throughout all segmental levels of the mouse lumbar spinal cord (15). Previous results from our laboratory indicate that interneurons of lamina VI and dorsal lamina VII across the lumbar enlargement of the adult cat do not spatially segregate based on the interneurons’ locomotor phase of firing (16). Kwan et al. (17) also showed through calcium imaging of the neonatal mouse ventral horn that there was no indication of rostrocaudal spatial clustering of interneurons with similar phase modulation during fictive locomotion (17), complementing results in the neonatal rat that showed no functional grouping of locomotor-related interneurons at the dorsoventral midline (18). In addition, a vast rostrocaudal spread of first-order spinal interneurons in the deep dorsal horn have been shown to synapse onto gastrocnemius motor pools in the mouse (19), demonstrating the breadth of premotor interneuron connectivity within the spinal cord.

We addressed these conflicting hypotheses by quantifying the activation times and spatial distribution of spinal interneuronal populations in the fully transected, adult, air-stepping cat. Going beyond analysis of single-cell activity, multiunit studies analyzing groups of simultaneously recorded single neurons were employed. Interest in population scale neural analysis of behavioral coding has brought to light the necessity for large-scale recordings due to the highly statistical relationship of ensemble activity to behavior (20–25). Of particular interest has been the study of groups of single units exhibiting similar firing dynamics during behavior, termed neural ensembles (26, 27). Therefore, we examined population ensemble activity and its relation to locomotion. Our findings provide greater insight into the contribution of single cells and population scale activity to patterned, rhythmic behavior through evidence that ensembles participate in alternating phases of stepping and are distributed throughout the lumbar enlargement.

## MATERIALS AND METHODS

### Animals and Experimental Procedures

Five adult domestic short hair female cats (2.4–2.9 kg) were used in this study. All animal care and procedures were approved by the Institutional Animal Care and Use Committee of Drexel University and were performed according to National Institutes of Health guidelines. All animals followed the same experimental protocol. First, a T11/T12 spinal transection was performed 22–23 days before the recording session to potentiate the air stepping induced by clonidine administration during the terminal procedure (16). Spinal transection and postprocedure care followed the laboratory standard procedures (16, 28). No locomotor training was provided to the animals at any point before the terminal experiment. On the day of the terminal recording experiment, three sets of surgical procedures were performed on the anesthetized animal: *1*) a laminectomy exposed the lumbar cord, *2*) bifilar EMG electrodes were implanted into seven muscles of each hindlimb (Table 1), and *3*) a mid-collicular decerebration preceded the discontinuation of anesthesia.

**Table 1.** Muscles recorded bilaterally

Muscle (Abbr)	Primary Function	Secondary Function
Sartorius anterior (SA)	Hip flexor	Knee extensor
Biceps femoris anterior (BFA)	Hip extensor	Knee extensor
Vastus lateralis (VL)	Knee extensor	
Biceps femoris posterior (BFP)	Knee flexor	Hip extensor
Gastrocnemius medialis (MG)	Ankle extensor	
Soleus (SL)	Ankle extensor	
Tibialis anterior (TA)	Ankle flexor	

### Surgical Procedures before Recording Session

Consistent with previously published experiments in our laboratory (16, 29), animals were initially injected with atropine (0.05 mg/kg im) and anesthetized with isoflurane (1.5%–3.5% in oxygen) supplied through an endotracheal tube. Heart rate, blood pressure, end-tidal CO_2_, tidal volume, arterial oxyhemoglobin saturation, respiration rate, and temperature were monitored and recorded every 15 min. Intravenous fluids were administered (20 mL/h) throughout the terminal procedure and dexamethasone (2 mg/kg iv) was given before the laminectomy to reduce spinal swelling. A spinal laminectomy removed the exposed spinous processes from sacral segment 1 rostrally toward lumbar segment 3 and the surrounding bone, leaving the transverse processes of each segment intact and exposing the spinal cord from segments L2–L7.

### EMG Electrodes Implant

Muscles of the upper and lower hindlimbs were exposed with two incisions. Seven muscles of each hindlimb (Table 1) were implanted with bifilar electrodes constructed with insulated multistrand stainless steel wires (AS 633; Cooner Wire, Chatsworth, CA). The electrodes were implanted into the body of the muscle and secured onto the fascia with sutures. Proper electrode placement was verified by stimulation of the electrode and observation of the resulting muscle’s twitches. Incisions were closed with sutures. A stimulating cuff electrode was implanted around the sciatic nerve to identify motor neurons that backfired at a short latency in response to electrical stimulation of the nerve.

Following the laminectomy and EMG electrode placement, the animals were transferred to a stereotaxic frame where the spinal vertebrae were securely clamped to the frame. Trunk skin flaps were used to form a mineral oil pool that prevented desiccation of the cord following opening of the dura. Roots were identified and used as anatomical landmarks of the lumbar segments. The pia was opened at planned recording sites to ease electrode insertion and prevent dimpling of the cord. A mid-collicular decerebration was performed and anesthesia was discontinued, as isoflurane has been shown to disrupt the activity of the spinal locomotor circuitry (30).

### Extracellular Recording Procedures

At ∼1 h postdecerebration, clonidine (500 µg/kg) was administered intravenously to prime air stepping (31). Once air stepping was controllably inducible through perineal stimulation, experimental recording sessions began. All of the recording trials were conducted on the right side of the spinal cord. Two 64-site microelectrode arrays (model A8×8-5mm-200-200-177, Neuronexus, Ann Arbor, MI) were inserted at or near the dorsal root entry zone to an approximate depth of 3,000 µm in two lumbar segments. The planar eight shaft arrays were inserted sagitally, i.e., in the rostrocaudal direction, so that the recording sites covered a range of 1,450 µm rostrocaudally and 1,450 µm dorsoventrally (from ∼1,500 to 3,000 µm deep) for each array. In our first experiment, one electrode array was statically placed in L7 while the other array was successively placed in rostral segments L3–L6. However, low neuronal yield at the L7 site required adaptation of the protocol for the remaining four experiments to static placement of one array in L3, whereas the other array was successively placed caudally in segments L4–L7.

The recording trials obtained for each pair of spinal locations consisted of: *1*) air-stepping trials involving a 5-s resting state followed by 50 s of air stepping (containing 40–60 step cycles) and a 5-s rest period, *2*) control rest trials (no perineal stimulation), and *3*) sciatic nerve stimulation trials (current amplitude at 1.3 times motor threshold, 1–2 Hz, 60 s, biphasic pulses with 100-µs duration for each phase) at rest. The rest and sciatic stimulation control trials as well as four to six air-stepping trials were executed at each set of unique recording location pairs.

Extracellular neural activity and muscle EMGs were recorded with a Tucker-Davis Technologies RZ2 system including 128 channels for recording multiunit activity and 15 analog channels for 14 EMGs and 1 sciatic stimulus recordings. Extracellular voltages were conditioned through a PZ2-128 preamplifier (Tucker-Davis Technologies, Alachua, FL) and bandpass filtered (300–4,000 Hz for single and multiunit data) before being sampled at 24 kHz for offline analysis. EMG voltages were amplified and bandpass filtered (10–5,000 Hz 4th order Butterworth, 10 K gain, Differential Amplifier Model 1700, A-M systems Inc., Carlsborg, WA) before being sampled at 12 kHz. Reference and ground were tied to a bone screw in the skull.

Following completion of the recording experiments, electrodes were dipped in DiO dye (Molecular Probes, Eugene, OR) and reinserted into recording locations for verification of stereotaxic measurements. The animals were then euthanized with an overdose of Euthasol (0.4 mg/kg iv). The lumbosacral spinal cord was fixed posthumously by soaking it in 4% paraformaldehyde solution for ∼20 min. The spinal cord from segments L2–S1 was then removed, fixed in a 4% buffered paraformaldehyde solution and refrigerated for at least 3 days. The cords were then blocked into segments, frozen, and sectioned in the transverse plane. Observation of the DiO-traced electrode tracts was used as in Ref. 16 to verify laminar location of the recordings.

### EMG and Stepping Characteristics Data Processing

All data processing and analysis for neuronal and EMG recordings were performed using software written in MATLAB (The MathWorks, Natick, MA).

Raw EMG voltages were high-pass (4th order elliptical, 100 Hz cutoff frequency, zero phase) filtered, full-wave rectified, and low-pass (4th-order elliptical filter, 15 Hz cut-off frequency, zero phase) filtered to develop linear activity envelopes. EMG burst onsets and offsets were identified with an algorithm based on the generalized likelihood-ratio test (32). The onsets were used to determine the start and end of the step cycle as well as the flexor and extensor phases of gait. A step cycle was defined as right soleus (ankle extensor) onset to consecutive right soleus onset.

Five criteria were used to test the quality of the stepping in terms of both left-right limb alternation and flexor-extensor alternation (16, 29): *1*) extensor duration ratio, *2*) flexor duration ratio, *3*) flexor phase ratio, *4*) cycle duration ratio, and *5*) left onset lag. The extensor duration ratio was calculated by dividing the right extensor burst duration by the left extensor burst duration and the flexor duration ratio was calculated by dividing the right flexor burst duration by the left flexor burst duration. These two ratios provided the relation between the burst durations of the extensor and flexor muscles of the left and right hindlimbs; both values would be 1.0 if the muscle burst durations were the same for both hindlimbs. The flexor phase ratio was calculated by dividing the right flexor onset phase (relative to the right step cycle) by the left flexor onset phase (relative to the left step cycle). This ratio described the symmetry in flexion onset time between the two hindlimbs. The cycle duration ratio was calculated by dividing the right step cycle duration by the left step cycle duration. For a 1:1 ratio in stepping between the two hindlimbs, this ratio would be 1.0. The left onset lag was defined as the phase of onset of the left extensor within a right step cycle and for symmetrical left-right stepping, this ratio would be 0.5. Significance for each of the ratios was evaluated using the *t* test with α = 0.05.

Frequency of stepping was determined as the average number of flexor and extensor bursts per trial. Frequency of stepping was tested for consistency across air-stepping trials using a *t* test (α = 0.05).

### Muscle Grouping Analysis

Cluster analysis (33) of onset and offset phases of all ipsilateral flexor and extensor muscles determined muscle groupings. First, the onset and offset burst times for each muscle were normalized to the phase of the step cycle based upon the right soleus burst onset. Each muscle’s onset and offset phases were clustered and outliers greater than 2 standard deviations from the mean of each cluster were removed. For a muscle pair to be grouped, the custom cluster technique required that the distance between the means of each muscle’s onset-offset phase cluster be less than the sum of the standard deviations times a constant (cFrac that was set at 1.25 in our analysis). This process was iterated through each ipsilateral muscle pair. We calculated the percentage of times that muscle pairs were clustered together. If significant groupings occurred in the majority of trials per experiment, the clustered muscles were considered to have equivalent onset and offset times.

### Neural Signals Data Analysis Overview

**Figure 1.**
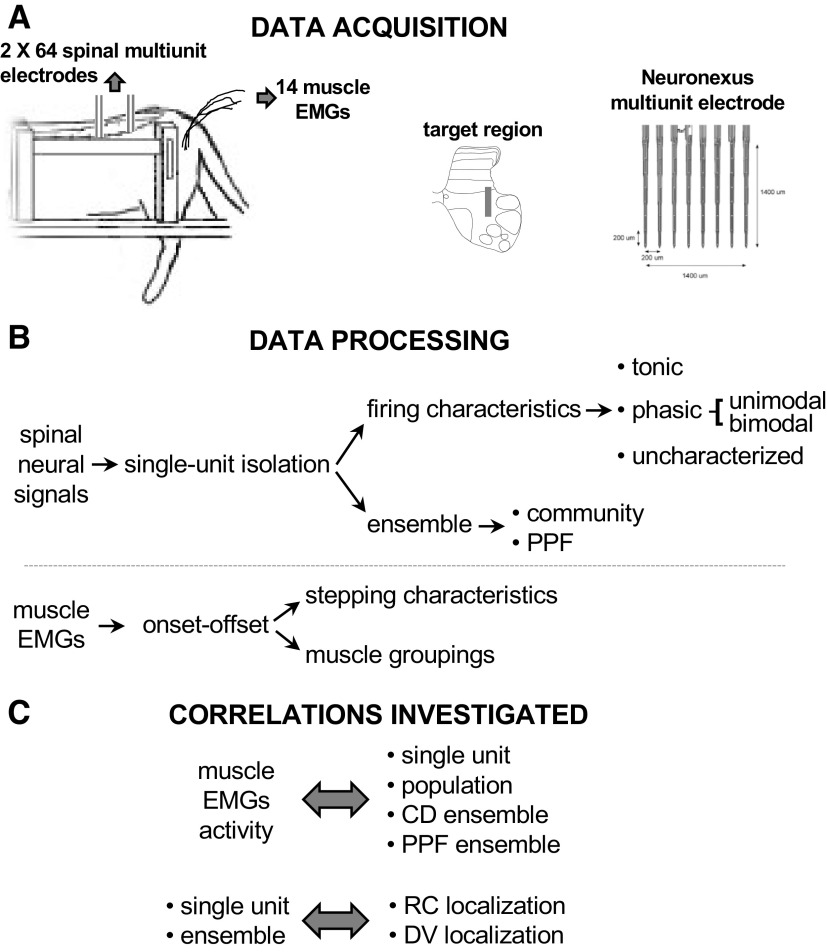
*A*: 64 channels of multiunit activity were collected from the spinal intermediate zone of decerebrated spinal cats, along with bilateral hindlimb muscle EMGs (7 muscles each limb). The geometry of the spinal recording electrode (image from Neuronexus website) covered an area of 1,400 by 1,400 µm along the rostrocaudal axis of the cord; two electrodes were inserted into the cord, one rostral (L3 or L4) and one caudal (L4–L7). *B*: spinal signals were processed to extract single units that were then analyzed to establish their firing characteristics (tonic or phasic (bimodal or unimodal) and ensemble memberships (either community detection ensemble (CD) or preferred phase of firing (PPF) ensemble). *C*: the relationships between muscle activity (mainly in term of burst onset/offset) and neural activity at the single unit level, population level or ensemble (CD or PPF) were investigated using correlation analyses (see text for details), the main hypothesis of functional neuronal organization was investigated by analysis the correlations between the single units’ phase of firing or ensemble memberships and their rostrocaudal and dorsovental locations.

Neural signals analysis used three approaches to explore different aspects of the neural activity (Fig. 1*B*). First, we analyzed single neuron spike times during locomotion. We then focused on the spike times of the entire population of simultaneously recorded neurons during locomotion. Finally, we broke down the populations into ensembles to classify spinal neural networks and their relation to rhythmic motor output. Units were classified into ensembles based on two methods: a community detection ensemble and a preferred phase of firing (PPF) ensemble. The community detection ensembles were grouped based on Humphries’ community detection algorithm (34), whereas the PPF ensembles were determined based upon the cells’ preferred phase of firing.

### Spike Sorting

Single neuron spike sorting was performed using UltraMegaSort2000 software (35). To be considered for analysis, single units had to meet the following criteria: *1*) greater than 5 spikes per step for at least 5 consecutive steps, *2*) greater than 200 spikes in a trial, *3*) contain less than 1.5% refractory period violations for a refractory period of 1.5 ms, *4*) signal-to-noise ratio greater than 1.5 (36), and *5*) not respond with a short latency to sciatic nerve stimulation.

Spike-triggered averaging methods were used to test for evidence of motor neuron antidromic backfiring in response to sciatic stimulation. If the averaged signal from that channel following sciatic nerve stimulation displayed a peak in activity above baseline within 1.4–3.4 ms after delivery of the antidromic stimulus, it was flagged as a motor neuron and removed from further analysis (37).

Due to our experimental protocol, it was highly probable that a single neuron was recorded from the same location over multiple trials. We thus tracked distinct neurons across trials using a Variational Bayesian Gaussian Mixture Model (VBGMM) method. The first three principal components of each action potential’s waveform shape were calculated for every unit recorded at the same electrode site. A VBGMM was fit to the matrix of the first three principal components to form an unbiased threshold for distinct neurons recorded across multiple trials (38, 39). Unlike *k*-means clustering, this method did not require an a priori solution to the number of clusters. It only required a value for the maximum number of clusters that could have been present (in our case, the number of trials recorded at the same location). Neuron membership of a unit was assigned to the group where the majority of that unit’s waveforms clustered.

### Single-Unit Firing Characteristics

Single units were classified according to their firing behavior during air stepping as tonically, unimodal phasically, or bimodal phasically firing units. Tonically firing neurons displayed constant firing throughout a step with very little variation in firing rate within and across the step. Phasically firing neurons displayed higher statistical probability of firing at a particular phase of the step cycle; one phase for unimodal phasic and two phases for bimodal phasic firing.

Neuron spike times were tested for tonic firing in 8 s of air stepping by analysis of the interspike interval histogram’s (ISI) coefficient of variation squared (40). A γ distribution was fit to the ISI (41, 42), with variables α and β estimated via maximum likelihood estimation (MATLAB function, gammafit). If the ISI histogram came from a γ distribution (2-sample Kolmogorov–Smirnov, goodness of fit test, α = 0.05), the coefficient of variation-squared value was taken as the 95% confidence interval of the γ distribution. The coefficient of variation-squared value is a good representation of the variation of spike times of a neural signal; the greater the coefficient of variation, the greater the variance in spike times over a trial and the likelihood that the unit’s firing is phasic. The lower the coefficient of variation, the less variation in firing times throughout the trial and the more constant the firing. To be considered tonically firing, a neuron’s coefficient of variation squared had to be less than 0.5. This threshold was chosen because the coefficient of variation squared is roughly equivalent to the inverse of the γ distribution order, with values between 2 and 4 best fitting regular spiking (40).

To determine whether a neuron’s activity was significantly modulated to stepping, its spike times were analyzed with circular statistics (16, 29, 43). A histogram of spike times was created from the relative phase (from 0 to 1) of spike timing during each step; 0 being the extensor onset time and 1 the onset of the next extensor cycle. The normalized spike times were plotted in a raster plot over all steps for greater than 5 consecutive steps of a trial and summed over 72 equally spaced bins throughout the locomotor cycle (44). In addition, 1,000 surrogate spike trains maintaining the same ISI histogram distribution were created for each neuron studied. Neurons were included for tuning analysis if their modulation histogram contained bins whose amplitude was larger than 3 standard deviations above the mean of the surrogate spike trains. Due to the cyclic nature of stepping, the resulting normalized histogram was analyzed for the magnitude (r), direction (ϕ), and angular deviation (s) of the mean resultant of activity (45). A neuron with a magnitude of 1 indicates firing at the same phase for every step, whereas a magnitude of 0 indicates no modulation of activity. The Rayleigh test was used to test the significance of unimodal modulation (α = 0.05) (46, 47). If a neuron was classified as significant for unimodal tuning, the direction of the resultant was considered its preferred phase of firing (from 0 to 1).

As the Rayleigh test of uniformity assumes a unimodal distribution, two tests were performed to distinguish unimodally tuned neurons from bimodally tuned neurons. The circular analog to the Gaussian distribution, the vonMises distribution, was fitted to the binned spike time histogram to assess whether the neuron’s modulation to the step cycle was unimodal. Maximum likelihood estimation was used to estimate the vonMises scale factor, κ, and the model was fit to the normalized data set using two parameters (κ and resultant magnitude of the preferred direction, r).

To classify the bimodally tuned neurons, a generalized vonMises distribution developed for circular bimodal distributions was estimated (48, 49) for each normalized histogram of neural firing. Maximum likelihood estimation of normalized spike times provided the estimates for the four parameters (u1, u2, k1, k2) of the generalized vonMises distribution. To be considered a bimodal distribution, the model needed to fit a number of criteria: *1*) scale factors (k1, k2) had to be sufficiently large (>0.1) so that peaks were relevant, *2*) ratio between the peaks’ amplitudes (lowest peak amplitude/greatest peak amplitude) had to be greater than 20% to ensure that both peaks were of relevant relative amplitude, *3*) the greatest trough height could not be more than 50% of the amplitude of the smallest peak height, to establish adequate distinction between peaks, *4*) local minima and maxima detection functions had to detect only two peaks and two troughs in the model (MATLAB, imregionalmin, findpeaks), and *5*) the separation statistic (the shortest distance between the peaks/the sum of the peak tuning widths) had to be greater than one to ensure that the peaks did not overlap.

Modality (unimodal vs. bimodal) was determined by a test that measured both the goodness of fit of the model to the data, while accommodating for the number of parameters used to create the model. The log likelihood ratio test compared the four parameters of the generalized vonMises model (u1, u2, k1, k2) to the two parameters of the restricted vonMises model (κ, r). If the full model (generalized vonMises) had a greater log likelihood than the restricted model (vonMises) (*P* < 0.05, llr test), a nonparametric test for goodness of fit determined whether the generalized vonMises model fit the data (Watson *U*^2^ test, α = 0.05). Finally, an omnibus test for circular uniformity determined whether the data were uniformly distributed throughout the step (Hodges–Ajne test, α = 0.05). After passing all criteria, the bimodal distribution’s two preferred phases were determined as the peak phases of the model.

#### Community detection ensemble determination.

The community detection algorithm, detailed in Humphries (34), was used to define ensemble group membership of simultaneously firing single units during air stepping. The algorithm identifies similarities between the firing of units, dividing them into ensembles sharing similar firing patterns but requires no a priori specifications of the number of groups to find and has only a single free parameter related to the timescale of the comparison between firing patterns to adjust.

Each air-stepping trial was broken down into 4-s data windows with a sliding overlap of 2 s. For each 4-s window, every spike train was convolved with a Gaussian kernel to provide a continuous spike density waveform. Humphries’ algorithm uses a modularity index as a measure of intragroup similarity of neural firing. To maximize the modularity index used to define ensemble participation, a selection of 10 evenly spaced Gaussian widths (the single free parameter to adjust in the algorithm) was chosen to iterate through to choose the smoothing width that maximizes the modularity index.

**Figure 2.**
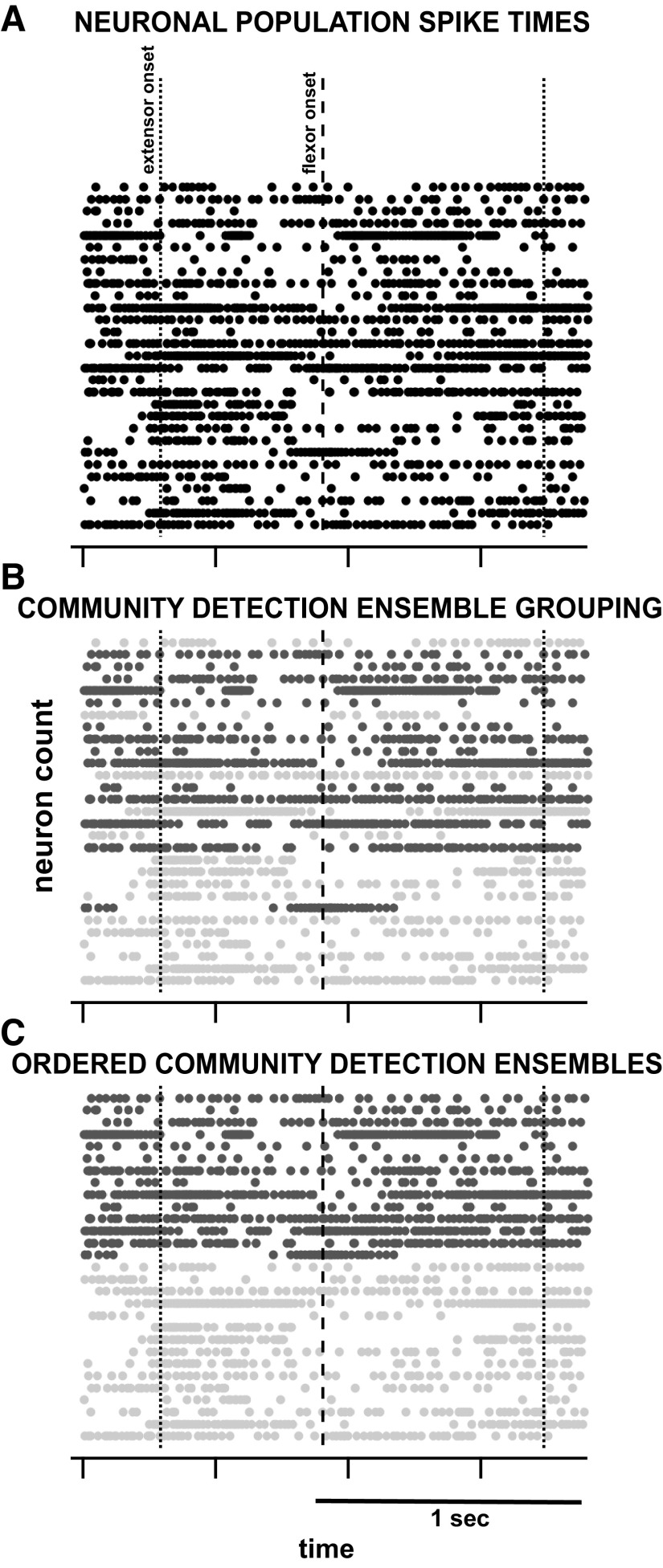
Example of community ensemble membership clustering. *A*: 1.5 s of spike times for 32 simultaneously active neurons. *B*: displays the same neurons as the top panel, labeled by their ensemble as grouped by the community detection algorithm. *C*: the reordered neurons according to their ensemble membership. Vertical bars indicate the EMG extensor (dotted) and flexor (dashed) onset times.

A similarity matrix of pairwise comparisons based on the cosine of the angle between spike densities was used in the clustering technique and provided a modularity index for every Gaussian width. In addition, a control modularity index was calculated from the average of 1,000 control datasets of shuffled ISI spike times, maintaining the same mean and variance of firing rates while eliminating neural correlations (50). The Gaussian width that maximized the modularity index beyond the control modularity index was used in ensemble grouping (mean width of 6.7 ± 3.5 ms, *n* = 529). Finally, the grouping matrix found to maximize the modularity index provided both the number of groups based upon iterative *k*-means clustering and group membership of each neuron to an ensemble (see example in Fig. 2).

In some instances, neurons within 4-s windows were not consistently assigned the same group membership across all windows of an air-stepping trial. To finalize the group membership in those cases, a similarity matrix of binary group membership per neuron was analyzed by minimizing the hamming distance between the binary rows of all groups and windows of a trial. The result provided a final determination of group size and membership to all neurons of a trial to an ensemble.

#### Preferred phase of firing ensemble determination.

Neurons whose unimodal, preferred phase of firing was significant within a trial were assigned to one of two ensembles based upon the preferred phase of firing. The first ensemble’s neurons had preferred phases in the first half of the step cycle (0–0.5), whereas the second ensemble-contained neurons whose preferred phase of firing was in the last half of the step cycle (0.5–1.0).

#### Correlations between the community detection ensembles and EMG motor output.

We examined each ensemble’s relation to motor output by correlating the spike density waveform (the Gaussian smoothed spike times of all neurons within an ensemble) to the envelopes of one ipsilateral flexor and extensor EMGs. EMGs were high pass filtered at 35 Hz (4th order, zero phase Butterworth), full wave rectified and low pass filtered at 1,000 Hz (4th order, zero phase Butterworth) to obtain the envelopes. As shown in Fig. 2, our results grouped only 2 ensembles per trial. Therefore, comparison between ensemble firing and EMG activation resulted in four quantities describing the correlation between each ensemble and the muscle for each trial; *ensemble 1* to flexor correlation, *ensemble 1* to extensor correlation, *ensemble 2* to extensor correlation and *ensemble 2* to flexor correlation. These four correlation results per trial were tested for mean differences across subjects and locations using a one-way analysis of variance test and a post-hoc multiple comparisons test including a Bonferroni correction for multiple comparisons (α = 0.05).

The correlation to muscle activity for the PPF ensemble was analyzed as with the community-based ensemble clustering method. Comparisons between the two methods provided a measure of how neurons with no preferred phase of activity related to locomotion may encode additional information about muscle activity.

#### Relationship between ensemble membership and single-unit firing characteristics.

We compared the preferred phase of firing of significantly tuned individual neurons to their community detected ensemble membership to examine the potential relationship between the more biologically relevant preferred phase of firing and the purely mathematical ensemble clustering. This was performed using the Watson Williams test (a circular analog to the 2 sample *t* test, α = 0.05) between the preferred firing phases of the units in each ensemble.

### Comparisons of the Correlations between the Different Neural Activity Modalities and EMGs

Correlations between hindlimb muscle EMGs and each of the processed neural signals (single unit, population, community detected ensemble and preferred phase of firing ensemble) were calculated. For each of the four neural data subsets, the spike times were binned (0.5 ms width) and convolved with a 100 ms Gaussian window to create a continuous spike waveform.

The single highest quality flexor and extensor EMGs for each hindlimb were used to respectively represent the flexion and extension phases of air stepping for each limb. EMGs were high pass filtered at 35 Hz (4th order, zero phase Butterworth), full wave rectified and low pass filtered at 1,000 Hz (4th order, zero phase Butterworth) before correlation to the continuous spike waveform.

For each trial, we calculated the Spearman correlation coefficient (exact permutation distributions, α = 0.05) between each neural processing subset and each of four hindlimb muscles (ipsilateral and contralateral flexor and extensor muscles). The correlation results were analyzed for differences in coefficients’ medians between left and right side stepping for each of the four neural processing subsets using the nonparametric Kruskal–Wallis test (α = 0.05) as well as post hoc multiple comparisons test including the Bonferroni correction (α = 0.05) (see results in Fig. 11). These tests were employed to detect any differences in the ability of neural data types to encode for locomotor output.

### Relationship between a Neuron’s Location and Its Firing Dynamics

A number of tests were employed to evaluate the relationship between a neuron’s location and its firing characteristics: *1*) a Mardia linear-circular correlation was performed between the individual neuron’s circular preferred phase of firing and its rostrocaudal location for significantly tuned neurons (α = 0.05), *2*) a Mardia linear-circular correlation was performed between the individual neuron’s circular preferred phase of firing and its dorsoventral location (α = 0.05), *3*) a linear correlation was calculated comparing the linear coefficient of variation squared (a measure of neural firing regularity) of each neuron to its rostrocaudal location (α = 0.05), *4*) a linear correlation was calculated comparing the linear coefficient of variation squared of each neuron to its dorsoventral location (α = 0.05), and *5*) the group membership of community-detected ensemble participation was compared with the neuron’s rostrocaudal and dorsoventral positions using a binomial logistic regression (main effect and interaction term in the regression model, α = 0.05) to detect spatial grouping for neurons of an ensemble.

## RESULTS

### Summary

Data from five cats were collected over 141 air-stepping trials, 34 rest trials, and 31 sciatic nerve stimulation trials. Two spinal locations were sampled in most trials, resulting in 265 recordings with 128 channels recorded simultaneously. On average, we collected 28 ± 9 air**-**stepping trials per subject (*subject 1*: 44, *subject 2*: 25, *subject 3*: 23, *subject 4*: 22, *subject 5*: 27). These trials had on average 7 ± 1 unique penetrations/locations per subject (*subject 1*: 9, *subject 2*: 5, *subject 3*: 7, *subject 4*: 7, *subject 5*: 6) forming 5 ± 1 unique recording rostral/caudal pairs (*subject 1*: 6, *subject 2*: 4, *subject 3*: 5, *subject 4*: 5, *subject 5*: 5). On average, we obtained 5 ± 2 air-stepping trials per unique recording pair (*subject 1*: 7 ± 3, *subject 2*: 6 ± 1, *subject 3*: 5 ± 1, *subject 4*: 4 ± 2, *subject 5*: 6 ± 1).

All recordings were conducted on the right side of the spinal cord between lumbar segments 3–7 (L3 = 95, L4 = 47, L5 = 37, L6 = 44, and L7 = 42 trials). The majority (81%) of recordings took place at a depth of 1,600–3,000 μm from the dorsal surface of the spinal cord. A significant percentage (17%) took place within 500 μm of that range (9%: 2,700 μm, 2.5%: 2,500 μm, 3,200 μm, and 3,500 μm) and the final 2% were recorded at a depth from 800 to 2,000 μm from the dorsal surface of the spinal cord. Finally, 231/265 recordings were collected at the medial border of the dorsal root entry zone (DREZ). The remaining trials were obtained at more medial positions that were chosen to avoid blood vessels present on the dorsal surface of the spinal cord (4 at 200 μm medial to the DREZ, 5 at 300 μm, 7 at 400 μm, 10 at 600 μm, and 8 at 900 μm).

### Air Stepping Showed Symmetry in Flexor/Extensor Burst Durations and Right-Left Hindlimbs’ Cycle Durations

The air-stepping trials included 6,781 steps across all experiments. Each trial had an average of 48 ± 20 consecutive steps (*subject 1*: 51 ± 13, *subject 2*: 40 ± 16, *subject 3*: 67 ± 23, *subject 4*: 43 ± 22, *subject 5*: 38 ± 13). Hindlimb muscle bursts were analyzed for left-right and extensor-flexor symmetry during air stepping using the five ratios described in materials and methods. The average extensor and flexor duration ratios per trial were not significantly different than 1.0 for any of the cats (*t* test, *P* > 0.05). The average cycle duration ratios per trial were not significantly different than 1.0 in any of the five experiments (*t* test, *P* > 0.05). The left to right onset lag ratio was not significantly different from 0.5 in only one of the five experiments (*t* test, *P* > 0.05); however, it was within 10% of 0.5 in the remaining experiments. The mean flexor phase ratio was not significantly different from 1.0 in two of five experiments (*t* test, *P* > 0.05) and within 11%–22% of equal phases in the remaining three experiments [*subject 1*: (1.07–1.16), *subject 2*: (1.23–1.44), *subject 3*: (1.23–1.6)]. Overall, these ratios indicate symmetry between extensor and flexor muscle burst durations and in the step cycle durations between the left and right side.

### Flexor and Extensor Muscles Grouped into Two Clusters with Muscles in Each Group Having Similar Burst Onsets/Offsets

**Figure 3.**
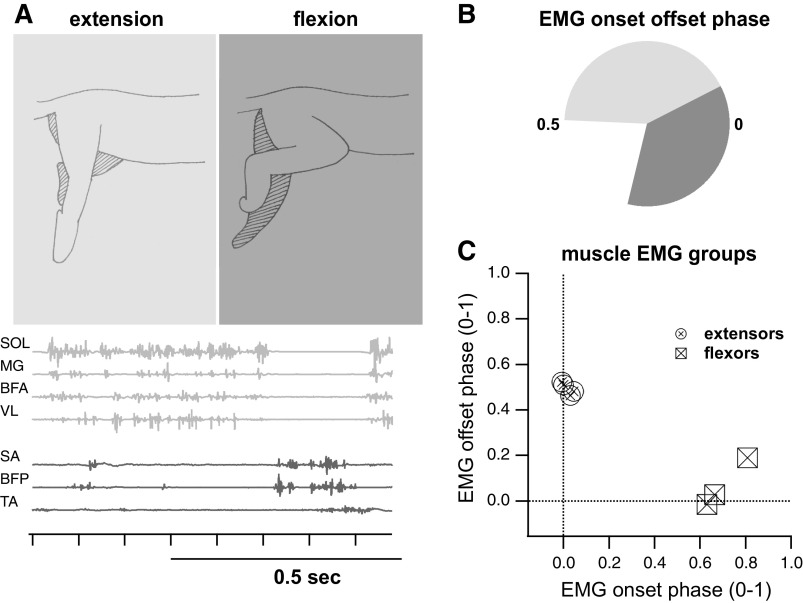
Muscle activity during air stepping and muscle groupings. The *top left* panel (*A*) is a pictorial representation of extension and flexion during air stepping. The corresponding EMG muscle bursts are shown in the *bottom left* plot over one step for each muscle recorded during air stepping and referred to by their abbreviations given in Table 1. The polar equivalent muscle burst durations are seen in the *top right* plot (*B*); the first half of the step cycle is taken up by the average extensor burst duration (of all 4 extensor muscles), whereas the last third of the step cycle contains the average flexor burst duration (between all 3 flexor muscles). The *bottom right* panel (*C*) shows that muscles clustered into two groups based on their burst onset and offset times. This tight grouping allowed us to use one extensor EMG and one flexor EMG as representative signals for each phase of air stepping.

The four right extensor activation times grouped together across all subjects and two of the three right flexor muscles grouped together in all subjects (Fig. 3). The right tibialis anterior showed the most deviation from the other flexor muscles’ burst times, beginning, and terminating ∼30% of the step cycle from the other flexor muscle onset and offset phases. This result was due to poor EMG electrode placement in 2/5 subjects and therefore, the TA was removed from consideration in all subjects.

Based on these results, the soleus (SL) activity was chosen to represent the extension phase of locomotion and the biceps femoris posterior (BFP) activity was chosen to represent the flexion phase of locomotion in 3/5 animals. In the remaining two animals, the sartorius anterior (SA) was used as the flexor instead of the BFP due to its greater signal quality, although both the BFP and SA had the same onset and offset times according to the EMG cluster analysis. Four EMG signals were processed per trial, the ipsilateral and contralateral representative flexor and extensor.

On average, right side extension occurred from 0 to 0.5 of the right step cycle and right side flexion occurred from 0.6 to 1.0 of the right step cycle. Midcycle coactivation of extensor and flexor activity was not present in any experiment and a gap in muscle activation from 0.55 to 0.6 was frequently present (3/5 experiments). The frequency of stepping per trial ranged from 0.9 to 1.2 Hz and was not significantly different from 1 Hz (*t* test, *P* > 0.05).

### Overview of the Single-Unit Results

**Figure 4.**
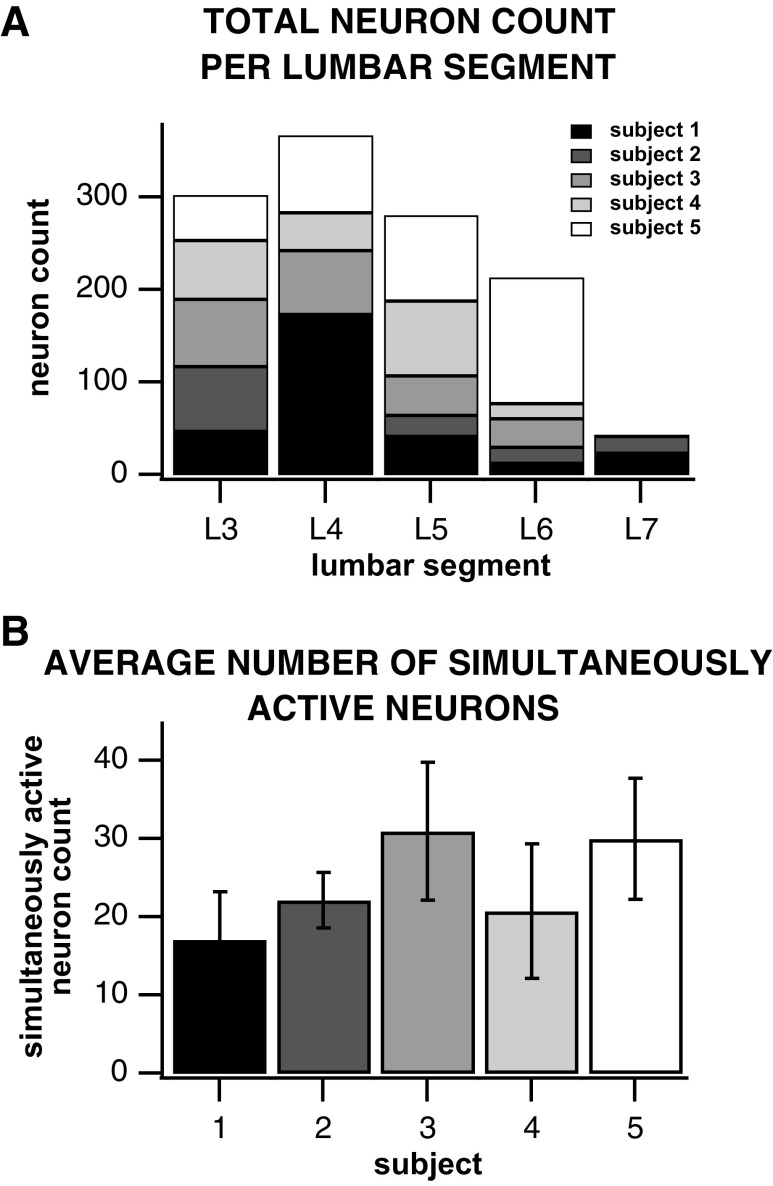
*A*: overview of the total number of neurons recorded during air stepping per subject and lumbar segment. *B*: mean and standard deviation of simultaneously recorded neurons per subject across all air-stepping trials.

We identified 3,291 single units in five cats. On average, each neuron was recorded over 2.7 trials, resulting in 1,207 distinct spinal interneurons recorded from the intermediate and ventral horn of lumbar segments 3–7. Figure 4 provides an overview of the number of single units isolated per lumbar segment and subject. The majority of single units were located in lumbar segments 3–6. There were greater than 18 simultaneously collected single units, on average, per air-stepping trial across all subjects and upward of 30, on average, in two subjects. We captured an upper limit of 45 concurrently active interneurons during a few air-stepping trials. In addition, we isolated 64/5,611 (1.1%) channels whose antidromic response to sciatic activation occurred within 1.4–3.4 ms from the sciatic nerve stimulus pulses delivered at an anatomical location (mid upper shank) that would backfire motoneurons innervating the lower hindlimb. These channels were considered as potentially containing signals from motor neurons and were not considered for analysis.

### About Half of the Interneurons Studied Showed Phasic Activity during the Step Cycle

**Figure 5.**
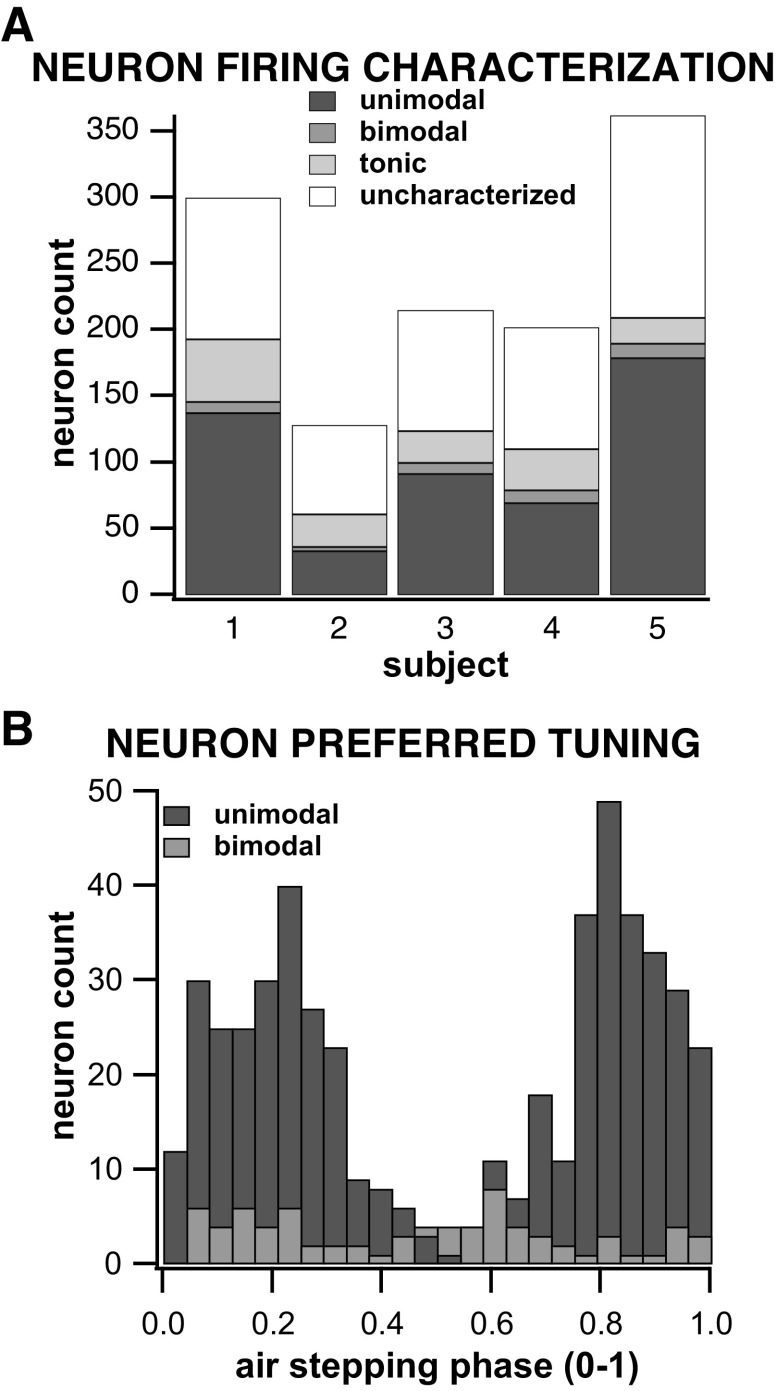
*A*: distribution of neuron count according to firing activity during air stepping per subject. The proportions for each type of neuronal activity pattern (tonic, phasic unimodal, phasic bimodal, uncharaterized) were consistent across all subjects. *B*: distribution of all bimodal and unimodal units’ preferred firing phases during a step.

About 46% (551/1,207) of the interneurons studied were phasically active during stepping with 509 showing a unimodal firing phase distribution and 42 showing a bimodal distribution (Fig. 5*A*). The mean preferred phase of firing of the unimodally tuned neurons was 0.97 ± 0.25 of the step cycle (Rayleigh test of uniformity; *P* < 0.05) (Fig. 5*B*). A small percentage of interneurons (12.1%, i.e., 146/1,207) showed tonic firing during locomotor activity (Fig. 5*A*), whereas the remaining 42% of the interneurons recorded could not be characterized as either phasically active (unimodal or modal) or tonic (Fig. 5*A*, uncharacterized).

### Community Detection Ensemble Algorithm Grouped Interneurons into Two Clusters in Most Cases

**Figure 6.**
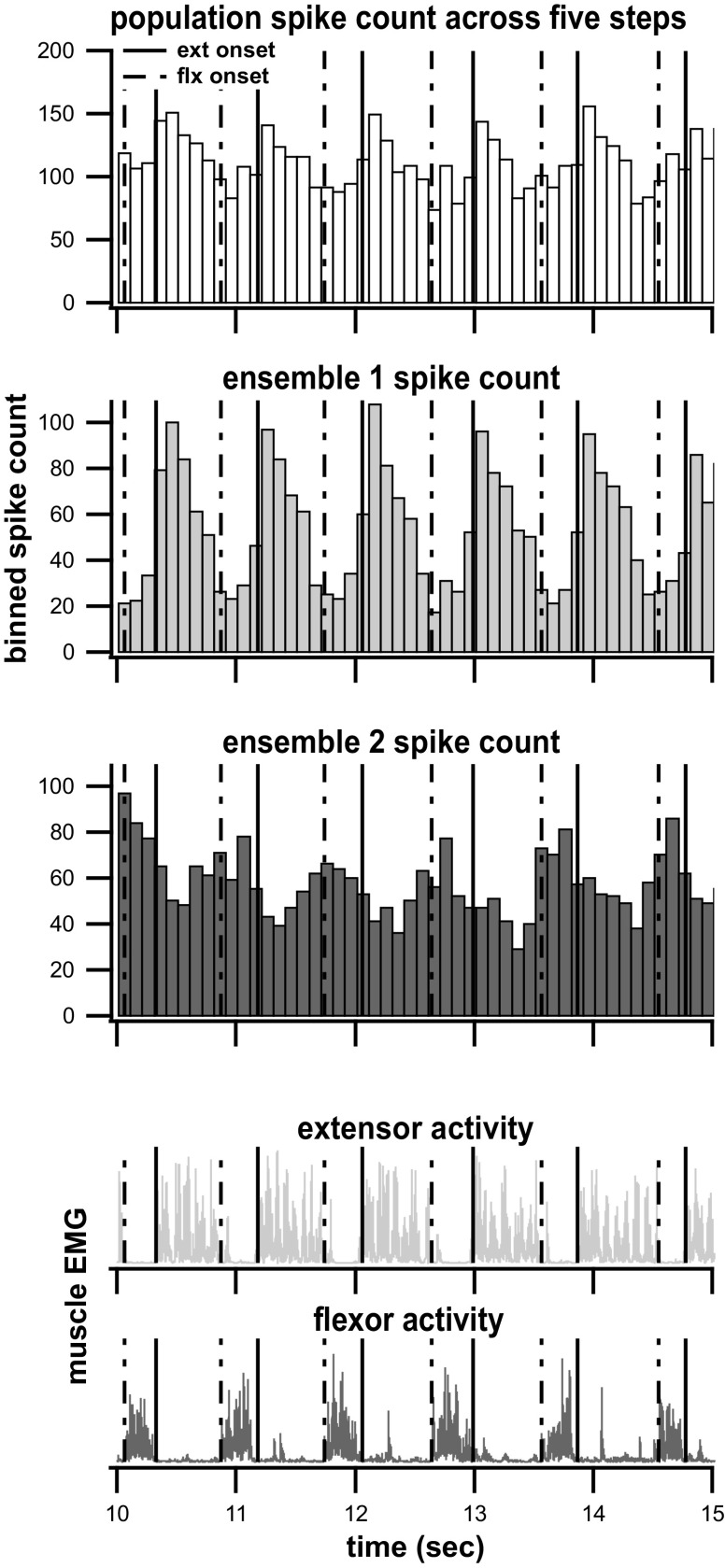
Community detection ensemble. The *top* plot shows an example of the binned spike count from the total population. The *second* and *third* panels are the ensemble spike counts, as determined via the community detection algorithm. Summed together, these ensemble spike counts would equal the top panel population values. The *bottom* two panels show the EMG envelopes of the representative ipsilateral flexor and extensor muscles across five steps. The solid vertical lines indicate right extensor onsets and the dashed vertical lines indicate right flexor onsets.

Community detection cluster analysis was performed on simultaneously recorded interneurons (563 interneurons recorded over multiple trials, 423 distinct ones) from 24 air-stepping trials (5 ± 1 trials per subject, 23 ± 8 neurons per trial, and 12 ± 4 neurons per ensemble) to obtain neural ensembles active during stepping. Almost all the trials (23 of 24) revealed two distinct neural ensembles (Fig. 6), whereas the remaining trial clustered the neurons into three ensembles.

### Community Detection Ensemble Activity Corresponds Strongly to Muscle Activation Patterns

Comparisons between ensemble firing and EMG activation resulted in four quantities for each trial: *ensemble 1* to flexor correlation, *ensemble 1* to extensor correlation, *ensemble 2* to extensor correlation, and *ensemble 2* to flexor correlation. The results show that out of 95 ensemble-to-muscle correlations found across 24 trials, 92 of them were significant (*P* < 0.05) indicating that 97% of the ensembles’ firing patterns correlate to EMG motor output. The average absolute value of the significant correlations between an ensemble and a muscle was 0.19 ± 0.13.

**Figure 7.**
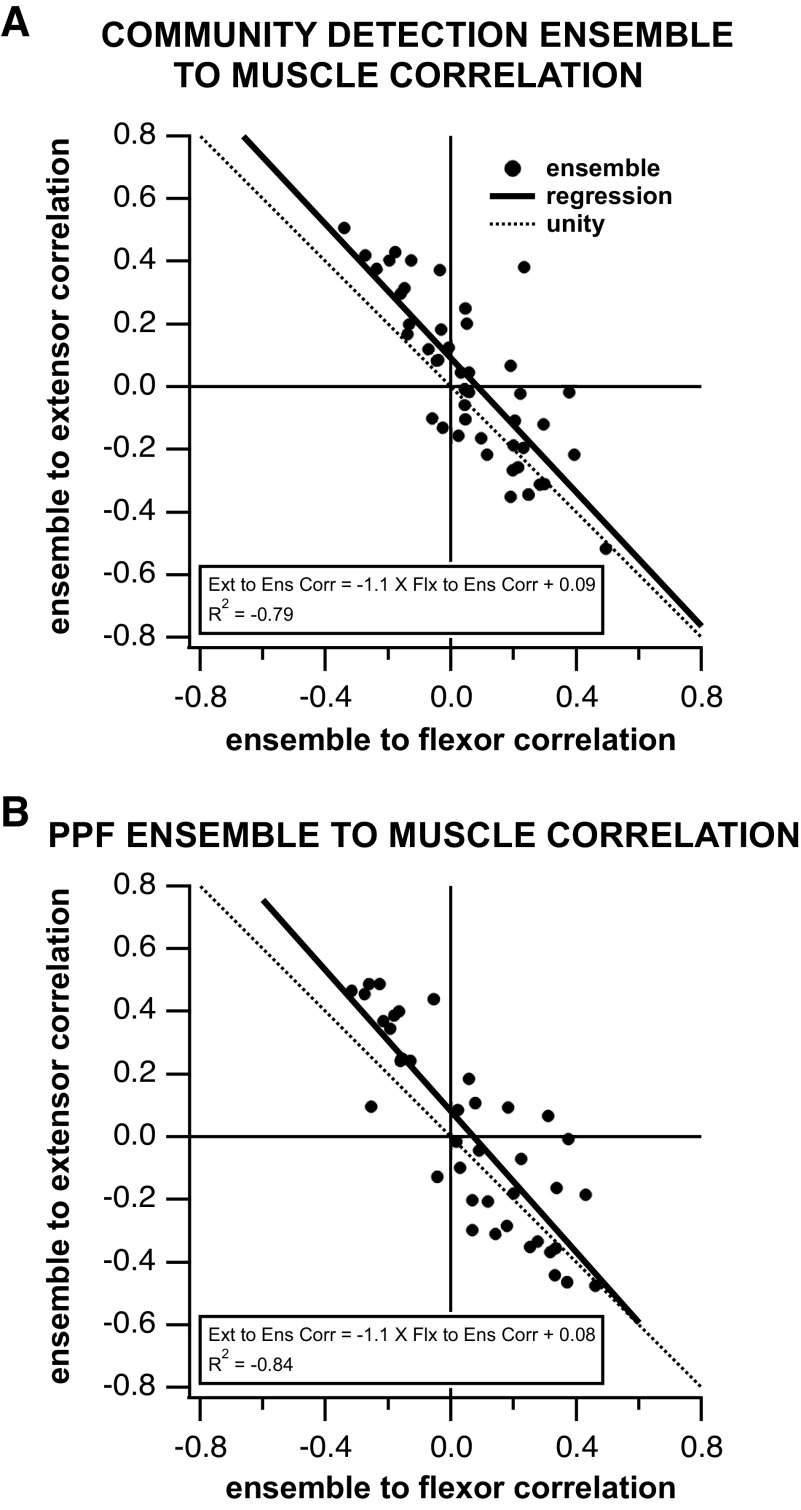
*A*: community detection ensemble to muscle correlation: Each ensemble was correlated to an ipsilateral flexor and an ipsilateral extensor EMG. Each point represents the correlations to the flexor (*x*-axis) and extensor (*y*-axis) muscles for a single ensemble. Only significant correlation values were included in the analysis (*n* = 45). The regression is nearly at unity and its location to the right of the unity line indicates that each ensemble’s positive muscle correlation is greater than its negative muscle correlation. *B*: preferred phase of firing (PPF) ensembles correlation to flexor and extensor EMGs. Analysis as for *A*.

A vast majority of ensembles (37/45, i.e., 82%) individually displayed a significantly positive correlation of their firing to one muscle group and a negative correlation to the opposing group. This result was further supported by the linear relationship between the ensemble to flexor correlations and the ensemble to extensor correlations (Fig. 7*A*). Sixty-seven percent (14/21) of the trials where the two ensembles had significant correlations to both muscles showed correlation values of opposite signs from each other for the correlations to flexor and extensor muscles, indicating that the majority of ensembles were correlated to opposing muscles during stepping.

**Figure 8.**
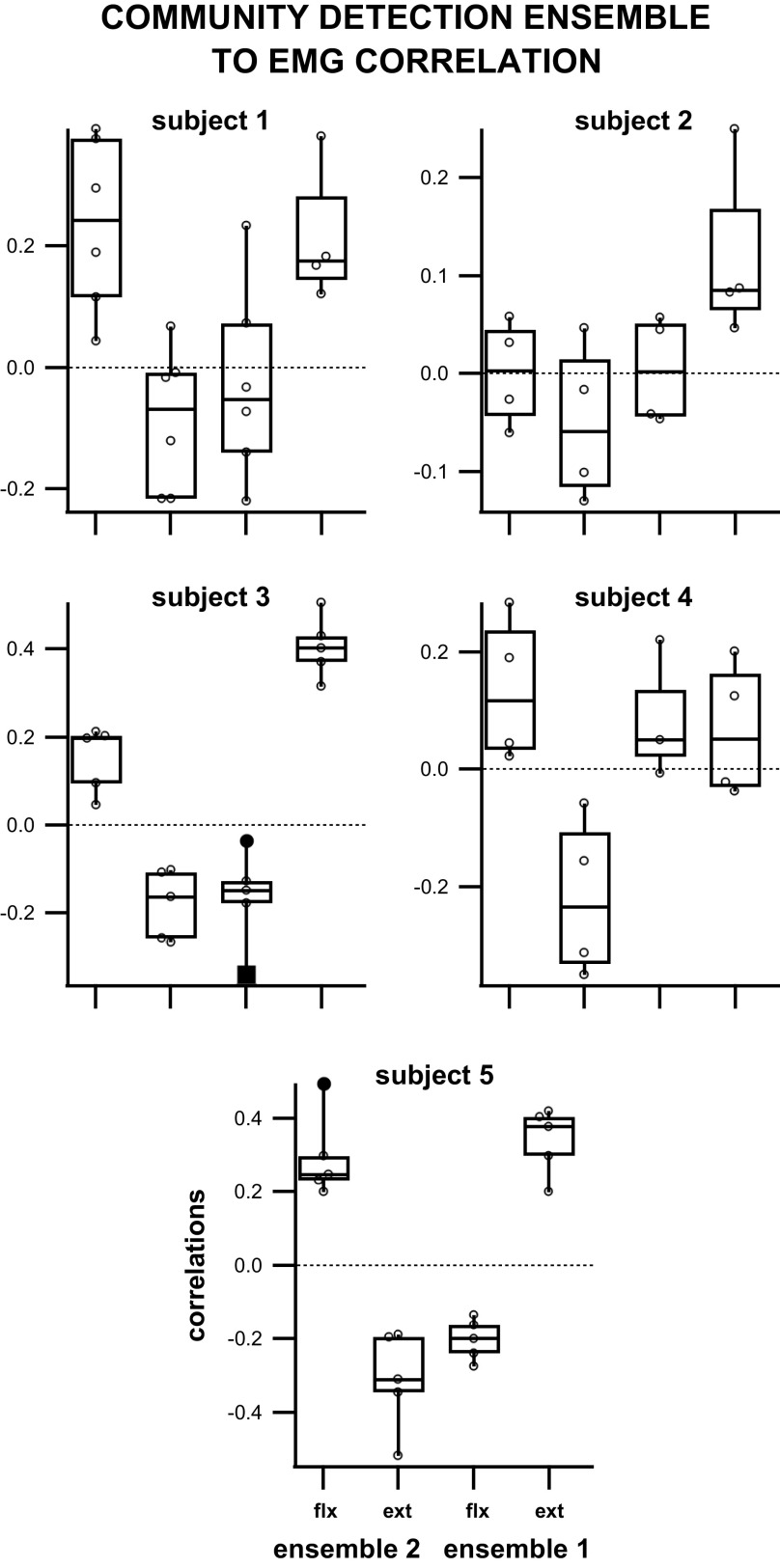
Box plot of correlations between community detection ensemble spiking and EMG of flexor and extensor muscles during air stepping per subject (correlation for the third ensemble of lowest neural membership for 1 trial of *subject 5* was not included). Only significant correlations (*P* < 0.05) were included (*subject 1* = 23 correlations; *subject 2* = 16 correlations; *subject 3* = 23 correlations; *subject 4* = 16 correlations; *subject 5* = 23 correlations).

**Figure 9.**
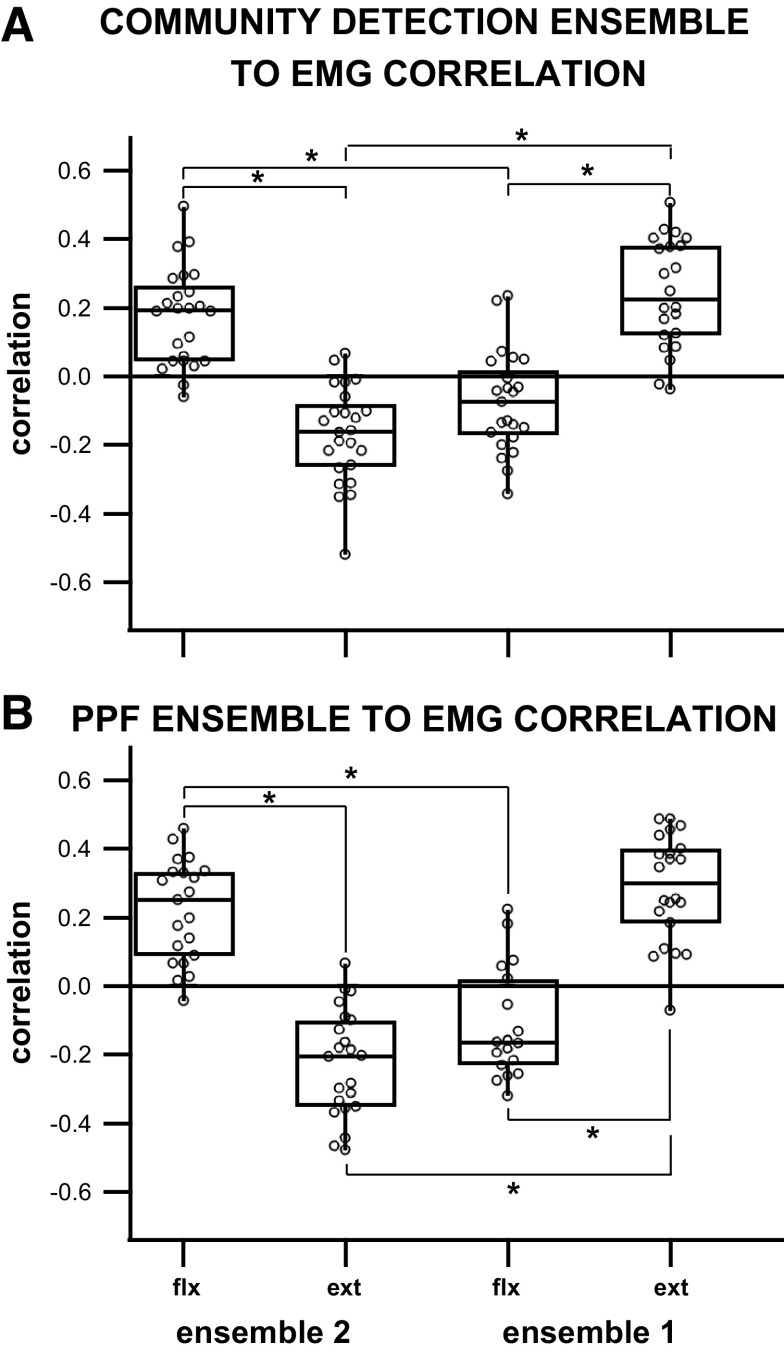
*A*: box plot of the correlations between community detection ensemble and EMG across all five subjects: each ensemble displayed a significant difference between correlation to flexor EMG and correlation to extensor EMG. In addition, interensemble comparisons showed a significant difference in the means of the correlations to the same muscle. *B*: box plot of the preferred phase of firing (PPF) ensemble to EMG correlations: each preferred phase of firing ensemble displayed a significant difference between correlation to flexor EMG and correlation to extensor EMG. In addition, interensemble comparisons showed a significant difference in the means of the correlations to the same muscle.

When analyzed per experiment/subject (Fig. 8), the means of the correlations between *ensemble 1* to the flexor muscle and of *ensemble 2* to the extensor muscle were shown to be significantly different from the means of the correlations between *ensemble 1* to the extensor muscle and of *ensemble 2* to the flexor muscle in 3/5 subjects. *Subject 4* displayed a significant difference between the mean of *ensemble 2* to flexor correlation and the mean of *ensemble 2* to extensor correlation, as well as a significant difference between the mean of *ensemble 2* to extensor correlation and the mean of *ensemble 1* to extensor correlation. *Subject 2* showed no significant difference between any ensembles to muscle correlation means. When analyzed for differences in the means across all subjects (Fig. 9*A*), there was a significant difference between the means of significant ensemble correlation to its respective muscles as well as a significant difference between the means of the two ensembles’ correlations to the same muscle [1-way analysis of variance, *F* = 42, df = 92, *P* = 7.1e-17; multiple comparisons tests with Bonferroni correction (α = 0.05)].

### Preferred Phase of Firing Ensembles Correlates Just as Strongly with Muscle Activation Patterns as the Community Based Ensembles

Comparisons between preferred phase of firing ensemble and EMG activation resulted in four quantities describing the correlation between the ensemble and the muscle: *ensemble 1* to flexor correlation, *ensemble 1* to extensor correlation, *ensemble 2* to extensor correlation, and *ensemble 2* to flexor correlation. The results showed that out of 92 ensemble-to-muscle correlations across 24 trials, 83 of them were significant (*P* < 0.05), indicating that 88% of the preferred firing phase ensembles correlate with the EMG motor output. The average absolute value of the significant correlation value between an ensemble and a muscle was 0.24 ± 0.14.

Eighty-four percent (36/43) of the preferred phase of firing ensembles individually displayed a significantly positive correlation to one muscle group and a negative correlation to the opposing group. This result was further supported by a linear relationship between the preferred phase of firing ensemble and flexor correlation to the preferred phase of firing ensemble to extensor correlation (Fig. 7*B*). Sixty-eight percent (13/19) of the trials, whose two ensembles showed significant correlation to both muscles, displayed opposite correlation values between muscles indicating that the majority of simultaneously recorded ensembles were correlated to opposing muscles during stepping.

**Figure 10.**
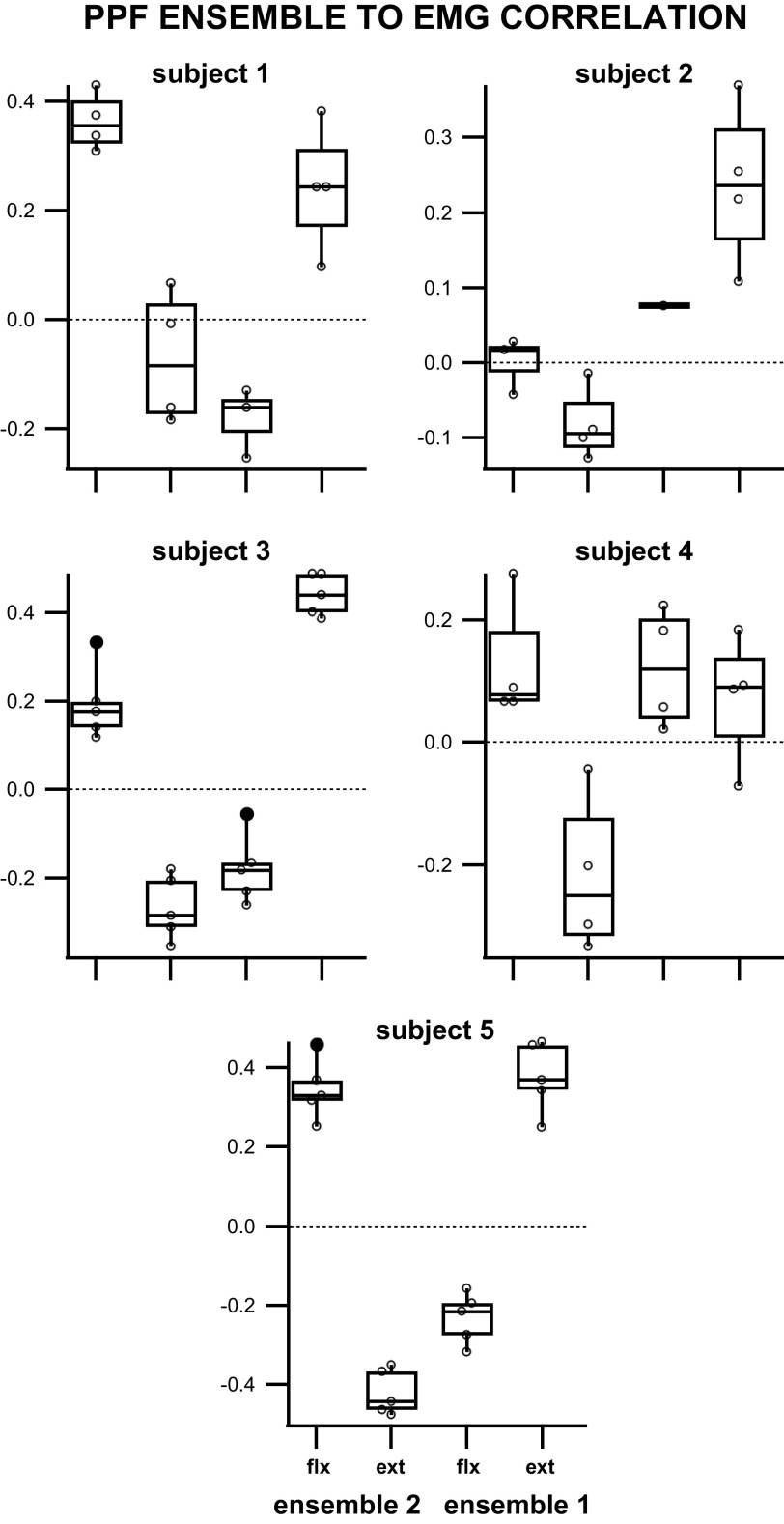
Box plot of the correlations between preferred phase of firing (PPF) ensemble spiking and EMG of flexor and extensor muscles during air stepping per subject. Only significant correlations (*P* < 0.05) were included.

When analyzed per experiment/subject (Fig. 10), preferred phase of firing (PPF) *ensemble 1* to flexor correlations and PPF *ensemble 2* to extensor correlations’ means were shown to be significantly different from PPF *ensemble 1* to extensor correlations and PPF *ensemble 2* to flexor correlations in 3/5 subjects (*subjects 1, 3, 5*). *Subject 2* displayed a significant difference between PPF *ensemble 2* to extensor correlation mean and PPF *ensemble 1* to extensor correlation mean. *Subject 4* showed a significant difference between PPF *ensemble 2* to extensor correlation and the other comparisons. When analyzed for mean differences in preferred phase ensemble to EMG correlation values across all subjects (Fig. 9*B*), there was a significant difference between the ensemble correlation to each muscle and a significant difference between the two ensembles’ correlations to the same muscle [one-way analysis of variance, *F* = 55, df = 82, *P* = 2.4e-19; multiple comparisons test with Bonferroni correction (α = 0.05)].

### Ensemble Firing Correlates Better with EMGs than Single Unit or Overall Population Firing

**Figure 11.**
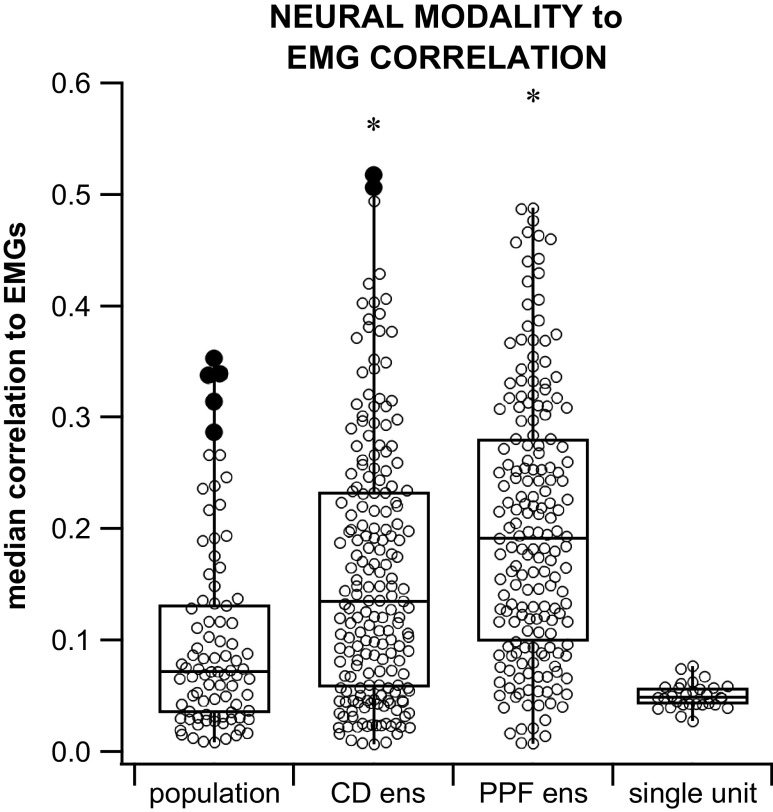
Box plot of the correlations between neural modality and EMG (±SD). Neural modalities include population, community detection ensemble (CD ens), preferred phase of firing ensemble (PPF ens) and single unit. Absolute, significant correlation values to ipsilateral and contralateral flexor and extensor muscle EMGs were grouped in these comparisons. Both the community detection and the PPF ensemble modalities showed a significantly greater median correlation to EMGs than the population and single unit median correlations. Both community detection ensemble and PPF ensemble correlation medians were significantly greater than the population and single-unit correlation medians (indicated by the *). The preferred phase of firing ensemble median correlation was significantly higher than the community ensemble median correlation but the population and single unit median correlations were not significantly different from each other [Kruskal–Wallis, df = 488, *P* = 4.2e-18, multiple comparisons test with Bonferroni correction (α = 0.05)].

When neural modalities (single unit, population, community detection ensemble, and preferred firing phase ensemble) were compared for differences in median correlation to all four muscles combined per subject, 4/5 subjects displayed significantly greater correlations between the preferred firing phase or community detection ensembles and EMGs than between single unit or population and EMGs. In *subject 1*, the preferred phase ensemble’s correlation to EMGs was significantly greater than the population and single unit’s correlations to EMGs but not greater than the correlation between community detection ensemble and EMG (which was only significantly greater than the single unit’s correlation to EMGs). *Subject 2* displayed a significantly greater median correlation of the preferred phase of firing ensemble to the single unit, but no other significant difference was detected. *Subject 3* displayed a significantly greater median correlation for both ensembles compared with the median correlation between population and EMGs, but no other significant difference was detected. In *subject 4*, no significance difference was detected. In *subject 5*, the preferred phase of firing ensemble’s correlation to EMGs was significantly greater than the population and single unit’s correlations to EMGs, but not greater than the correlation between community detection ensemble and EMG (which was only significantly greater than the population’s correlation to EMGs). Overall, results among subjects were consistent in that, in general, both ensemble correlation medians were greater than the population and single unit correlations and that the single unit correlation medians were typically the lowest correlation of all modalities (Fig. 11).

### Single Units’ Firing Dynamics Show No Relationship with Rostrocaudal or Dorsoventral Locations

**Figure 12.**
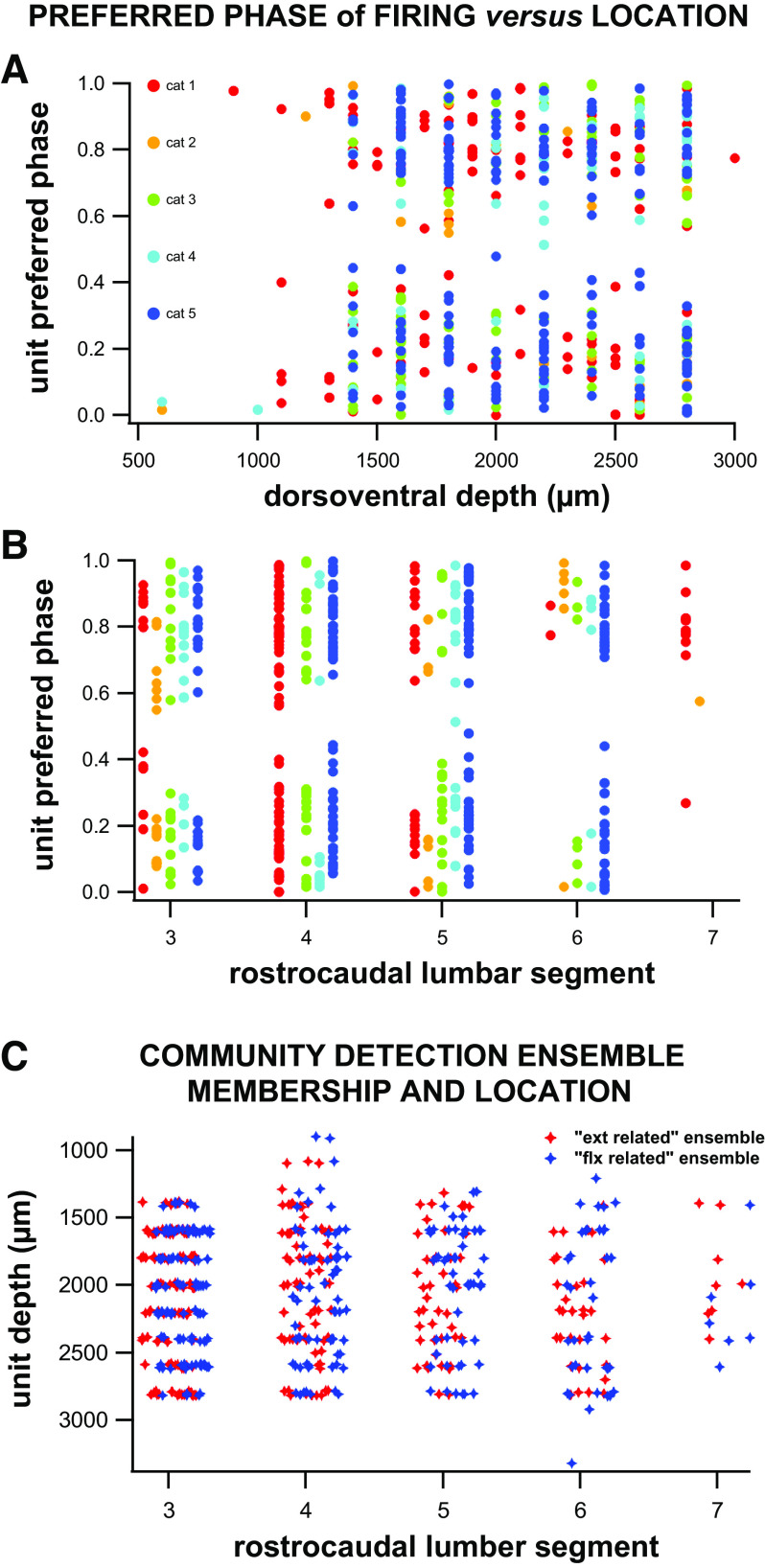
Relationships between the units’ preferred phase of firing and their depth (*A*) or rostrocaudal location (*B*). Preferred phase of firing showed no significant correlations to either depth (Mardia circular-linear correlation; ρ = 0.05, *P* = 0.58, *n* = 484 unimodally tuned neurons) or rostrocaudal location (Mardia circular-linear correlation; ρ = 0.08, *P* = 0.24, *n* = 484 unimodally tuned neurons). *C*: the community detection ensemble membership (for either extensor or flexor-related ensembles) as a function of rostrocaudal location and unit depth. Logistic regression showed no correlation between a unit’s membership and its location (see text for details).

There was no correlation between the preferred phase of firing of a single unit and its location along the rostrocaudal axis (measured as lumbar segment) (Mardia circular-linear correlation; ρ = 0.08, *P* = 0.24, *n* = 484 unimodally tuned neurons). When analyzed for each individual, there was significance in one subject (2) between the preferred phase of firing of single units and the lumbar segment; however, no significance was detected in any other case (Table 2 and Fig. 12*A*). In addition, there was no correlation between the coefficient of variation squared per single unit to its rostrocaudal lumbar segment (Pearson correlation; ρ = 0.02, *P* = 0.52, *n* = 1,207 neurons).

**Table 2.** Neural correlation between unit’s location and PPF

Test: correlation	Subject	Correlation	Significance (*P* < 0.05)	Relationship
R/C-PPF	1	0.18	0.12	No
R/C-PPF	2	0.46	0.03	Yes
R/C-PPF	3	0.04	0.93	No
R/C-PPF	4	0.23	0.24	No
R/C-PPF	5	0.04	0.88	No
D/V-PPF	1	0.11	0.47	No
D/V-PPF	2	0.35	0.14	No
D/V-PPF	3	0.27	0.04	Yes
D/V-PPF	4	0.18	0.39	No
D/V-PPF	5	0.04	0.85	No

D/V, dorsoventral; PPF, preferred phase of firing; R/C, rostrocaudal.

When analyzing for the relation between preferred phase of firing and dorsoventral depth of the unit, only *subject 3* showed a significant correlation between the dorsoventral depth of its neurons and its preferred phase of firing, but no significance was detected in any other subject (Table 2 and Fig. 12*B*) or overall for all units (Mardia circular-linear correlation; ρ = 0.05, *P* = 0.58, *n* = 484 unimodally tuned neurons). When the coefficient of variation squared was correlated to dorsoventral depth, there was a negligible correlation (Pearson correlation; ρ = −0.08, *P* = 0.0095, *n* = 1,207 neurons).

Finally, we tested for a relationship between community detection-ensemble membership and individual interneuron’s rostrocaudal and dorsoventral locations and found no significance between a unit’s location and community detection ensemble membership (binomial logistic regression, *P* > 0.5 for rostrocaudal and dorsoventral location main effects and interaction term and Fig. 12*C*).

## DISCUSSION

Our study shows that a large proportion of the interneurons from the more lateral edge of laminae V–VII of the L3–L7 segments are significantly tuned to the locomotor step cycle. Many of these cells are tuned to either flexion or extension, with interneurons tuned to either phase of the step cycle and few interneurons firing during the silent muscular activation period occurring between these two phases. This relationship between interneuron firing and the flexion/extension phases of locomotion is further supported by our neuronal assembly analysis that grouped units into two ensembles of spinal interneurons correlated to the opposing phases of the step cycle without a priori knowledge of those phases. Finally, interneurons with different phases of firing appear to be distributed throughout the cord, as we found no evidence of rostrocaudal or dorsoventral grouping of interneurons based upon their activation during a particular phase of locomotion or community detection ensemble membership.

### Neural Populations Tuned to Flexion and Extension Phases of Air Stepping

We found that interneurons of the intermediate and ventral horn of the lumbar spinal cord are active during both phases of the step cycle. These findings are in agreement with previous studies of the intermediate and ventral horn of the lumbar cord in the neonatal rat (14, 18, 44), the neonatal mouse (17) and the adult cat (16, 51, 52). In our study, single neuron preferred tuning phases were distributed between 0–0.3 and 0.6–1, which roughly translate to the first two-thirds of the extension phase and the flexion phase of stepping (Figs. 3 and 5). In addition, the quiescent period of single-unit firing from 0.3 to 0.6 matched the period of low/near-silent muscle activity occurring at the transition from stance to swing in the air-stepping preparation. This silent period of interneuronal activity started at about the last third of the extensor burst and probably lead to the early silencing of extensor activity, with both silencing possibly coming as a result of the lack of force feedback from ground force reactions present in overground locomotion but absent in air stepping (53). Interestingly, this early termination of extensor muscle activity before the flexors’ onset is not observed in the spinal intact real or fictive locomotion preparation (54, 55), even though sensory feedback is totally absent in the fictive preparation. Early silencing of the extensor bursts is also not observed in locomotor trained spinal cats walking on a treadmill (54). The early silencing of extensor bursts in our preparations may be due to a combination effect of spinalization, absence of locomotor training that reinforces sensory pathways (56, 57), and the absence of force feedback, which is critical in maintaining extensor activity in the absence of supraspinal input (58–60).

Contrary to motoneurons that are active throughout the corresponding period of muscle activity in fictive locomotion (55), single units do not typically fire during the whole period of muscle activation (see Ref. 16) but only during part of the period. As a result, single-unit firing to muscle activity correlations were poor especially compared with population or ensemble activity correlations to muscle activity (see Fig. 11). The poor concordance between the periods of interneuronal activity compared with the period of ensemble motoneuronal activity (i.e., whole muscle EMGs) during locomotion further argues for the need to study population activity of both interneurons and motoneurons in trying to elucidate the role of interneurons in the production of locomotor activity and might partially explain why muscle activity correlated much more strongly with population activity in our study than with single-units activity.

The identification of two ensembles of interneurons tuned to opposing locomotor phases is consistent with results in neonatal mouse fictive locomotion where genetically identified V1 interneurons divided into two groups of phasically bursting cells whose firing was 180° out of phase with each other (61). These neural ensembles may be participants in Graham Brown’s half center model (62). Demonstration of mutual inhibition between the constituents of those neural ensembles would provide evidence in favor of this hypothesis. Locomotion occurs through flexion and extension, which are out-of-phase with one another. As a result, we found that units or ensembles were significantly correlated to both phases of the step cycle, although correlations were typically higher for the positive correlation, i.e., the phase of locomotion where both neural and muscle activity were maximal.

The simple division of the units’ firing pattern into two clusters by the community detection ensemble algorithm neatly matched with the groupings of our seven recorded muscles into a flexor and extensor groups. We previously showed that those muscles similarly clustered into a flexor and extensor groups for spinalized cats walking on a treadmill (63), although (64) divided the activity of 19 muscles into 7 clusters for intact and spinalized cats using a similar approach. If these additional synergies are maintained in the spinal air-stepping cat, results in the frog demonstrating the presence of interneurons tied to muscle synergies’ activation (65) would predict that our interneuronal population should divide into more than two basic groups. More extensive recordings of muscular activity in the spinal air-stepping cat would be required to firmly establish whether additional synergies are expressed in this preparation, although another hypothesis is that our interneuronal activity mostly reflects the activity of the flexor/extensor rhythm generator layer of the mammalian central pattern generator (CPG) (66, 67). The period of low interneuronal activity corresponding with the silent period of muscle activity would argue against this hypothesis for a clock circuit composed of neurons connected in a mutual inhibition fashion, although activity of the flexor cycle could occur only after a period of inactivity of the extensor cycle of the clock. A more discriminating validation approach would be to evoke different motor behaviors such a flexion withdrawal, cross-extension or scratching in the decerebrate spinal cat and observe whether the neural activity remains organized as a flexor-extensor group for the rhythmic behavior and collapse into a single group tied to flexion or extension for the nonrhythmic flexion withdrawal and cross-extensor behaviors.

### Potential Roles of the Neuronal Ensembles

Although we know that the area of the spinal cord where we recorded contains commissural interneurons with roles in left-right alternation (68), the greater correlations between neural activity and the ipsilateral muscle activity (as opposed to the contralateral muscle activity) suggest that our interneurons are involved in ipsilateral sensorimotor activity. In addition, the regression line of ensemble correlation to EMGs (Fig. 7) to the right of unity shows that our ensembles’ positive correlations are greater than their negative correlations. This result suggests that the neural ensembles provide excitatory drives to their respective flexor and extensor motor neurons, which fits with the behavior of the pattern formation level of the two-level CPG model (66, 69), although they may also reflect activity of the rhythm generator level, as discussed above. Although we do recognize the prevalence of inhibitory circuitry involved in premotor flexor and extensor alternation (70–72), our results suggest that the interneuronal ensemble active during a particular phase has an overall excitatory drive to its respective motor population, although the possibility of some inhibitory connections to the opposing motor population may not be ruled out at this point. In addition, although the overall drive may be excitatory, the possibility that the drive from an interneuronal ensemble to a motoneuronal population is a concurrent mix of inhibition and excitation remains (73, 74).

### Spatial Distributions of Stepping-Related Neurons

The overall lack of spatial segregation between neurons tuned to opposing phases of the step cycle (Table 2) provides evidence that there are no gross rostrocaudal or dorsoventral groupings of neurons within the lumbar enlargement of the mature spinal cord. These findings are based upon neural firing characteristics including the preferred phase of firing, ensemble memberships and the units’ coefficient of variation squared. These results support a number of studies in the mouse, rat and turtle (18, 75, 76). Although there might very well be a spatial grouping of locomotor related spinal interneurons, the cell genetic types rather that their firing characteristics may be the delineating factor (61, 77). This observation is also confirmed by studies of H1 genetically identified interneurons that have firing patterns associated with the flexor and extensor phases despite sharing the same genetic markers (14). Overall, these results suggest that interneurons may be clustered based on genetical development patterns, but functional roles may subdivide following the initial localization within the cord.

### Biological Relevance of Mathematical Spike Time Clustering Algorithm

The community detection method of neural ensemble determination is based upon maximizing the intragroup similarity metric of similar spike times. This mathematical clustering technique allows for adaptable group numbers and requires no a priori knowledge of the neural system being studied. The vast majority of our results indicate that this mathematical clustering technique detected two ensembles without any input parameters regarding the biological behavior, whereas each ensemble was significantly tuned to an opposing phase of the locomotor step cycle. Not only do these ensembles reflect the EMG clustering results but the neural constituents thought to encode for this rhythmic activation are significantly related to the motor output in 4/5 subjects.

Furthermore, this relationship between ensemble activity and motor activity is significantly greater than the relationship between each individual neuron to motor output and the activity of the entire population of neurons to the rhythmic behavior. The significance and usefulness of population scale neural analysis is also indicated by 97% of interneurons being participants in locomotor-related ensembles while only 42% of neurons are significantly tuned to the step cycle. Our finding that nearly half of the single units were tuned to the locomotor step cycle has been repeatedly found in studies of the single unit relation to stepping (44, 75). The importance of population scale analysis has been extensively referenced (20, 25, 78, 79) and is now supported for spinal interneuron encoding of the rhythmic alternation of flexion and extension during air stepping.

## GRANTS

This study was funded by National Institutes of Health (NIH) Grants NS110605, NS055976, and EB012855.

## DISCLAIMERS

The funding sources had no involvement in study design, data collection, analysis, interpretation, or reporting.

## DISCLOSURES

No conflicts of interest, financial or otherwise, are declared by the authors.

## AUTHOR CONTRIBUTIONS

C.M., D.P.K., and M.A.L. conceived and designed research; C.M., D.P.K., A.J.K., and M.A.L. performed experiments; C.M., D.P.K., and M.A.L. analyzed data; C.M. and M.A.L. interpreted results of experiments; C.M. and M.A.L. prepared figures; C.M. and M.A.L. drafted manuscript; C.M., D.P.K., A.J.K., and M.A.L. edited and revised manuscript; C.M., D.P.K., A.J.K., and M.A.L. approved final version of manuscript.
